# IgY antibodies/Cysteamine: simple and effective methodology for electrochemical detection of SARS-CoV-2 S*-*protein

**DOI:** 10.1590/0074-02760250074

**Published:** 2025-10-20

**Authors:** Ariamna Gandarilla, Yonny Romaguera-Barcelay, Juliane Corrêa Glória, Luciana Freire, Taisa Farias, Jessica Feitosa, Carlos Anzola, Luís André Morais Mariuba, Walter Ricardo Brito

**Affiliations:** 1 Universidade Federal do Amazonas, Central Analítica Multidisciplinar, Laboratório de Bioeletrônica e Eletroanalítica, Manaus, AM, Brasil; 2 Fundação Oswaldo Cruz-Fiocruz, Instituto Leônidas e Maria Deane, Manaus, AM, Brasil

**Keywords:** IgY antibodies, electrochemical immunosensor, SARS-CoV-2 S-protein, cysteamine monolayers

## Abstract

**BACKGROUND:**

The outbreak of severe acute respiratory syndrome coronavirus 2 (SARS-CoV-2) infections was a serious disease that spread rapidly around the world and led to a state of global health emergency. During the pandemic, millions of deaths were notified as result of the progression of the disease to a serious condition. Research into the development of diagnostic tests was very important for the identification and control of new cases.

**OBJECTIVES:**

In this work a label-free electrochemical platform was developed for sensing of SARS-CoV-2 S-protein.

**METHODS:**

The S- antibodies (IgY type) from egg yolk were immobilised though stable bonding onto screen-printed gold electrodes surface, which was previously modified with self-assembled monolayers of cysteamine (Cys). The analytical performance of the devices was followed by differential pulse voltammetry after incubation in various concentrations of S-protein.

**FINDINGS:**

The electrical response exhibited a linear behaviour from 10 to 1000 ng mL^-1^ [with limit of detection (LOD) of 6.2 ng mL^-1^]. Also, we confirmed that our method is more sensitive than an enzyme-linked immuno-sorbent assay (ELISA), which was conducted with the same molecules (antibody and antigen) (500-4000 ng mL^-1^, with LOD = 235 ng mL^-1^). The immunosensor was selective for S-protein detection, and no significative changes were registered by differential pulse voltammetry in presence of SARS-CoV-2 N-protein. Tests on saliva samples recorded similar results to S protein standards.

**MAIN CONCLUSIONS:**

The developed immunosensor showed good performance and selectivity, therefore, it can be an alternative method for coronavirus disease 2019 (Covid-19) detecting in saliva samples.

Coronavirus disease 2019 (Covid-19) is a disease caused by the severe acute respiratory syndrome coronavirus 2 (SARS-CoV-2), which affected the population of most countries from 2020 to the present. The first cases appeared in Wuhan city, Hubei province, China, and were spread around the world, causing approximately 776 million of cases and 7.1 million of deaths. Different laboratories around the world have worked hard to develop effective vaccines against the disease, which was a crucial strategy to reduce morbidity and mortality. Different platforms for vaccine manufacturing have been used, each with its own advantages and disadvantages. Currently, the evolution of cases to severe stages has decreased due to high vaccination rates, with 13.6 billons of doses administered.^1^ ^,^ ^2^

Rapid detection of the virus is an important factor for the control and effective treatment of this disease. Classic methods are used for diagnosis, such as quantitative real-time reverse transcriptase-polymerase chain reaction (RT-qPCR) and enzyme-linked immunosorbent assay (ELISA).^3^ ^,^ ^4^ However, these methods are expensive, use complexes equipment, certified laboratories and must be carried out by qualified professionals.^5^ On the other hand, rapid tests for SARS-CoV-2 were an approach under development for point-of-care Covid-19 diagnosing, which are based on lateral flow immunochromatographic strips, coated with gold nanoparticles conjugated to antibodies or antigens. The assay detected IgM (sensitivity of 57% and specificity of 100%) and IgG (sensitivity of 82% and specificity of 100%).^6^ In the case of the antigen detection test (Ag-RDT), the reported sensitivity was variable, meaning that people who test negative may still be infected. A study for evaluate the performance of *CerTest* and *Panbio* Ag-RDT showed that both tests are specific (100%) but presented low overall sensitivity (53.5% for *CerTest* and 60.0% for *Panbio*).^7^ ^,^ ^8^ In this sense, it is possible developing alternative detection methods for SARS-CoV-2 diagnosis, like electrochemical immunosensors, due to offer some advantages: high sensitivity and specificity, the manufacturing process is relatively simple, use less expensive materials and equipment and easy execution.^9^

SARS-CoV-2 presents four principles proteins, including spike protein (S-protein), with molecular weight of 180 kDa and constitute by approximately 1300 amino acids. It has the function of recognising receptors and cell membrane fusion. That function makes it an important biomarker and possible target analyte to be detected by the recognition element present in the immunosensors.^10^ ^,^ ^11^ Some authors reported biosensors for SARS-CoV-2 S-protein determination, where different strategies were used for the device fabrication. The results showed low detection limits, high selectivity and good stability.^12^ ^,^ ^13^ ^,^ ^14^ ^,^ ^15^ ^,^ ^16^ ^,^ ^17^ The use of self-assembled monolayers (SAMs) is a methodology that allows immobilising molecules onto electrode surfaces. SAMs are formed by functionalised alkanethiols (like HS(CH_2_)nNH_2_), where the sulphur end can react chemically with gold surfaces forming a stable bond, and the NH_2_ group sited at the other end can be designed for a covalent bond with biomolecules (*e.g.*, antibodies). Specifically, the cysteamine (Cys), is a linking compound widely used for this porpoise when the substrates are gold electrodes.^9^ ^,^ ^18^ ^,^ ^19^ ^,^ ^20^ ^,^ ^21^

In this paper we fabricated an electrochemical immunosensor using Cys SAMs, which allowed the S-protein antibodies (S-Ab) immobilisation onto the surface of screen-printed gold electrodes (SPGE) for subsequent determination of SARS-CoV-2 S-protein.

## MATERIALS AND METHODS

*Reagents* - Cys, bovine serum albumin (BSA), N-(3-dimethylaminopropyl)-N’-ethylcarbodiimide hydrochloride (EDC), N-Hydroxysuccinimide (NHS), potassium hexacyanoferrate (III), carbonate-bicarbonate buffer of pH 9.60, tween 80 solution, glucose (Glu), potassium chloride, anti-chicken HRP antibody and artificial saliva were purchased from Sigma-Aldrich (USA). Potassium hexacyanoferrate (II) trihydrate, sulfuric acid (98%), and phosphate buffer saline (PBS) of pH 7.00 were from Merck (Germany). ELISA blocking buffer and One Step TMB Linear were from Scienco Biotech (Brazil). The aqueous solutions were prepared using Milli-Q water. The biomolecules (S-protein and S-antibody from SARS-CoV-2) were buyer from Ezscience (Manaus, Brazil), being the antibodies immunoglobulins Y type from egg chicken. The production process of SAR-CoV-2 N-protein (used in the specificity test) were described in previous work of our research group.^22^

*Apparatus and characterisations* - A PGSTAT128N instrument (Methrohm Autolab) was used to make electrochemical measurements, with NOVA software, 2.1.7 version. Gold electrodes (SPGE) (Methrohm Dropsens, Reference 220AT) were used as conductive substrate. These electrodes have a gold working electrode (4 mm of diameter), a gold auxiliary electrode, and silver electrode, which is considered a pseudo-electrode.

The modification with Cys SAMs was followed by Fourier-Transform Spectroscopy (FTIR), and the spectrums collected in an Agilent spectrophotometer, Cary 630 model (USA), coupled with attenuated total reflectance module (ATR). The scanning range was from 3500 to 650 cm^-1^, at 150 scan and resolution of 4 cm^-1^. Also, contact angle (CA) measurements were made after and before SAMs formation, using a digital microscope (Dino-Lite plus). Milli-Q water (5 μL) was added to the surface of bare and modified SPGE, and the droplet behaviour observed for 120 s at 22 ± 1ºC. The CA values were obtained in triplicate using the Image J software.

*Preparation and performance of the immunosensor* - First, the SPGE was washed with isopropyl alcohol, followed by Milli-Q water and finally dried using a nitrogen gas stream. For the SAMs formation, 8 µL of Cys (0.1 mol L^-1^) were dropped onto the working electrode (WE) and incubated for 18 h at 25ºC. Simultaneously, the carboxylic groups of the S-Ab were activated with EDC (0.1 mol L^-1^) and NHS (0.05 mol L^-1^) solution overnight. Afterwards, 8 µL of activated S-Ab (0.4 µg µL^-1^) were deposited onto WE for 4 h. Next, to avoid non-specific bindings during the antigen detection, a blocking with 0.1% BSA (previously activated with EDC/NHS) was carried out during 1 h. For determination of the target analyte, the S-Ab/Cys/SPGE immunosensor was incubated during 1h in different concentrations of S-protein, from 0.1 to 1000 ng mL^-1^, prepared in PBS solution. All the incubation steps were performed in a humid chamber to avoid evaporation of the solutions. The electrochemical characterisation of the assembly immunosensor steps, as well as the target analyte detection were followed by differential pulse voltammetry (DPV) in solution of 0.005 mol L^-1^ [Fe(CN)_6_]^4-/3-^+0.1 mol L^-1^ KCl, between -0.2 and 0.4V, at 25 mVs^-1^.

The specificity of the immunosensor for S-protein determination was tested for DPV in presence of other protein of SARS-CoV-2. For that, a solution containing S-protein (500 ng mL^-1^) + SARS-CoV-2 N-protein (500 ng mL^-1^) were prepared. No clinical samples were tested using this approach. However, an experiment was carried out using artificial saliva (solution prepared 1:10 v/v in PBS) enhanced with different concentrations of S-protein (10-1000 ng mL^-1^), and the electrochemical response of the immunosensor was measured by DPV in 0.005 mol L^-1^ [Fe(CN)_6_]^4-/3-^ + 0.1 mol L^-1^ KCl.

*Indirect ELISA test* - 96-well plates (SPL immunoplate) were sensitised by adding 50 µL of different concentrations of protein S (from 10 to 4000 ng mL^-1^) diluted in coating buffer (0.05 mol L^-1^ sodium carbonate and sodium bicarbonate), in triplicate for each concentration. The plate was then incubated in a humid chamber for 16 h at 4ºC. After this, blocking was performed by adding 150 µL of ELISA blocking buffer to each well and incubating the plates in a humid chamber for 2 h at 37ºC. Subsequently, the blocking buffer was discarded and 0.25 µg/well of S-Ab (IgY type) diluted in blocking buffer were added, incubating for 1 h under the same blocking conditions. After this, four washes were carried out with 200 µL/well of buffer (0.01mol L^-1^ phosphate buffer saline + 0.1% tween80), followed by incubation under the same conditions previously described with the anti-chicken HRP secondary antibody diluted in blocking buffer 1:10000. Later, the revelation was performed by adding 50 µL of One Step TMB Linear to each well and incubating away from light for 10 min, followed by the addition of 1 mol L^-1^ sulfuric acid solution to interrupt the reaction. Finally, the optical density (OD) was read at 450 nm in a spectrophotometer (LMR-96 model, Loccus, Brazil).

## RESULTS AND DISCUSSION

**Fig. 1:**
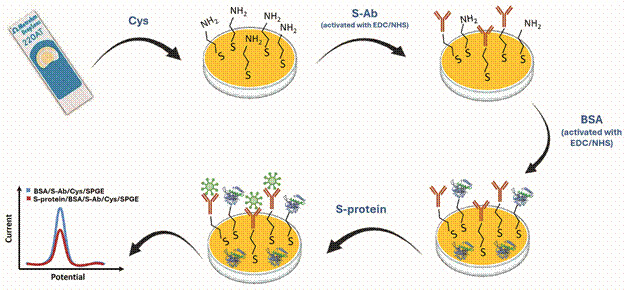
representation of the immunosensor assembling steps and detection of severe acute respiratory syndrome coronavirus 2 (SARS-CoV-2) S-protein.

The SAMs formation using Cys was the strategy selected to create functional groups onto the gold substrates that allowed subsequent S-Ab immobilisation (Fig. 1). This stage was successfully carried out and as expected, the thiol group interact with the gold WE and formed a stable bond.

**Fig. 2:**
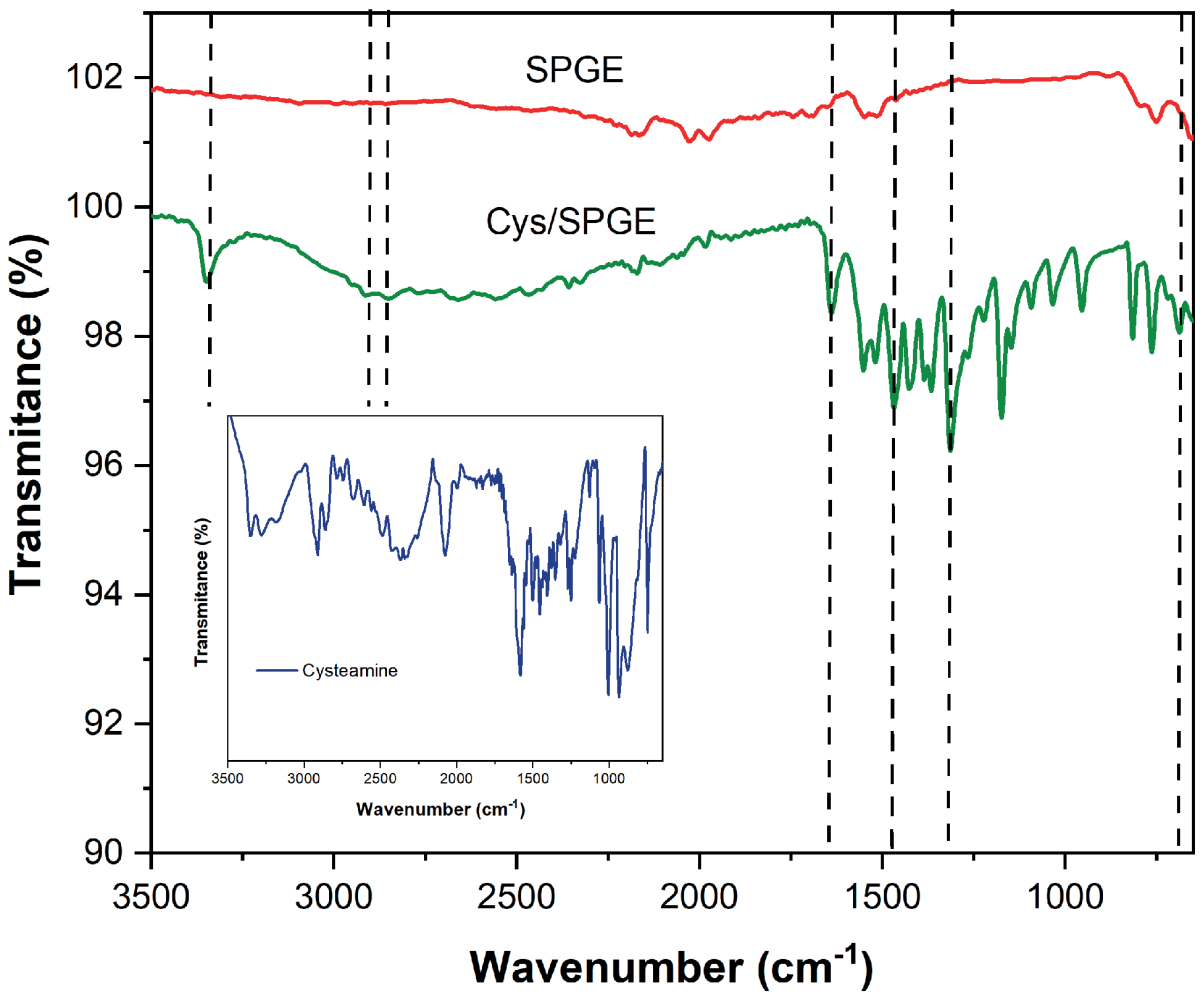
fourier-transform spectroscopy spectra collected after and before modification of screen-printed gold electrodes with cysteamine monolayers.

The spectroscopic characterisation after and before modification surface with Cys SAMs is observed in Fig. 2. As expected, the FTIR spectra of bare SPGE did not register any signal in the region of the collected spectrum. In contrast, the Cys/SPGE spectra confirmed the presence of Cys in the electrode surface, with a peak at 3348 cm^-1^ corresponding to N-H stretching. Two low intensity peaks at 2916 and 2951 cm^-1^ are characteristic of the C-H bond. The bands at 1639 cm^-1^, 1468 cm^-1^, 1318 cm^-1^, and 686 cm^-1^ are related to C = N group, C-N bend, C-C stretch and CH_2_ rocking, respectively. These results are correspondence with the band recorded by Cys sample (Fig. 2 inset) and the reported in other works.^23^ ^,^ ^24^ ^,^ ^25^ ^,^ ^26^

Studies of surface wettability play an important role in understanding many physical, chemical, and biological processes. Wetting is commonly characterised by the contact angle, which is defined as the angle between the tangent to the liquid-vapour interface and the solid surface at the three-phase contact line. The most common method of measuring the contact angle involves the use of low-magnification optical devices to observe the image of the droplet or bubble.^27^ ^,^ ^28^

**Fig. 3:**
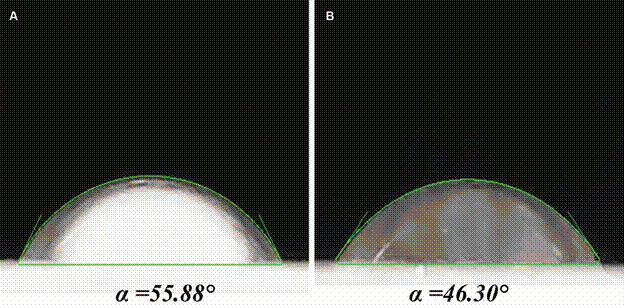
images obtained for water droplet after 120 s (A) Bare screen-printed gold electrodes and (B) cysteamine/screen-printed gold electrodes

The contact angle differences obtained after and before the modification are presented in Fig. 3. The results for the bare SPGE (contact angle = 55.88º) and Cys/SPGE (contact angle = 46.30º) suggest that both surfaces are hydrophilic [values for classification: hydrophilic (< 90º), hydrophobic (> 90º), or superhydrophobic (> 150º)].^29^ Also, a small increase of the hydrophilic properties was noticed for the electrode modified with Cys SAMs, due to the terminal NH_2_ groups in the HS(CH_2_)_2_NH_2_ structure, which gives it these properties. Similar results were reported by other authors for gold electrodes.^18^ ^,^ ^30^ ^,^ ^31^

**Fig. 4:**
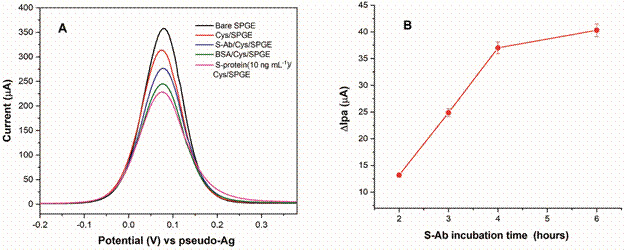
(A) differential pulse voltammetry response of the immunosensor assembling steps in 0.005 mol L-1 K3[Fe(CN)6]/K4[Fe(CN)6] + 0.1 mol L-1 KCl. (B) optimisation of S-antibody incubation time obtained by differential pulse voltammetry technique (standard deviation for n = 3)

*Electrochemical performance of BSA/S-Ab/Cys/SPGE* - The electrochemical response after each immunosensor modification step is presented in Fig. 4A, where is evidenced a voltametric profile with highest values of anodic peak current (Ipa = 357.57 µA) for the bare SPGE. After SAMs formation, was observed a noticeable decrease of the Ipa values (313.58 µA) due to surface blockage, hindering the passage of the redox probe ([Fe(CN )_6_]^4-/3-^) to the electrode. This behaviour has already been widely reported by different works that use this methodology.^18^ ^,^ ^30^ ^,^ ^31^ ^,^ ^32^ Prior to antibody immobilisation, the carboxylic groups of S-Ab were activated using EDC/NHS, with the aim of achieving a successful covalent bond to the amino groups present in Cys/SPGE. As expected, the presence of IgY type biomolecules with high molecular weight (~180 kDa) prevents the electrons flow and leads to current decrease (Ipa = 276.59 µA). In the next modifications, with BSA (Ipa = 244.97 µA) and S-protein (Ipa = 228.43 µA), the same behaviour was noticed, due to antigen-antibody specific binding.

Tests for optimising the incubation time in S-Ab solution were performed and until 4 h, the voltametric response registered by DPV technique exhibited a significant increase in ∆Ipa (∆Ipa = Ipa _(before S-Ab)_ - Ipa _(after S-Ab)_) values (Fig. 4B). After that, the increase is less noticeable, which is why 4 h was chosen as the appropriate time for the antibody immobilisation step.

**Fig. 5:**
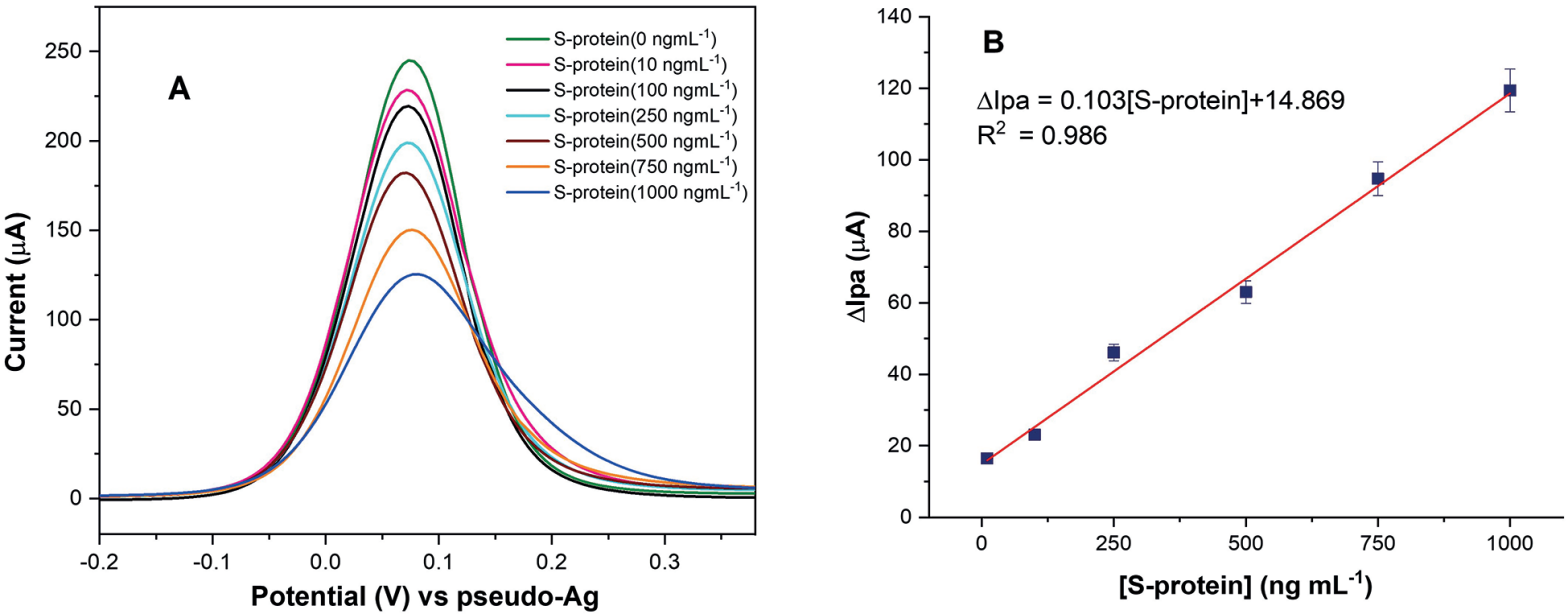
(A) electrochemical performance of the immunosensor obtained by differential pulse voltammetry in 0.005 mol L-1 K3[Fe(CN)6]/K4[Fe(CN)6] + 0.1 mol L-1 KCl. (B) calibration curve plot (S-protein concentration versus anode peak current variation) (standard deviation for n = 3).

The determination of SARS-CoV-2 S-protein using the BSA/S-Ab/Cys/SPGE immunosensor was carried out by DPV technique after incubation in different concentrations of antigen (0.1, 10, 100, 250, 500, 750 and 1000 ng mL^-1^) prepared in 0.1 mol L^-1^ PBS. In Fig. 5A the DPV profiles are represented, and the Ipa values decrease after each incubation in S-protein solution. At higher concentrations of S-protein, a greater number of antigens bind to the antibodies present in the sensor, leading to the passivation of the surface, which prevents the [Fe(CN)_6_]^4-/3-^ passage and consequently lower electrical currents are registered. For constructing the calibration curve, the ∆Ipa (µA) was plotted against the S-protein concentration (Fig. 5B). The graph shows a linear response in the 10-1000 ng mL^-1^ concentration range, with an R^2^ = 0.986. The LOD was 6.2 ng mL^-1^ (calculated as 3× standard deviation of blank divided by the slope of the calibration curve),^33^ indicating that there is adequate recognition between Ab-Ag.

**Fig. 6:**
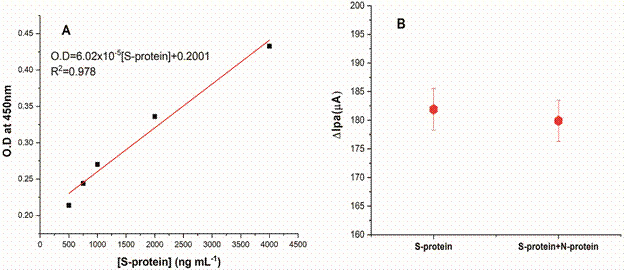
(A) calibration curve obtained by indirect enzyme-linked immunosorbent assay (ELISA) [optical density (OD) vs S-protein concentration] and (B) differential pulse voltammetry measurements in [Fe(CN)6]4-/3-+ KCl after incubation of the immunosensor in S-protein + N-protein solution (standard deviation for n = 3).

For comparison the results obtained by the immunosensor with a classic method for antigens detection, a study using indirect ELISA was conducted. Fig. 6A evidenced the linear behaviour from 500 to 4000 ng mL^-1^, with R^2^ = 0.978 and LOD = 235 ng mL^-1^. For lower S-protein concentrations, the ELISA results did not present good linearity. In this sense, we can affirm that the S-Ab/Cys/SPGE immunosensor reported here is more sensitive. Besides, to confirm the specificity of BSA/S-Ab/Cys/SPGE, a preliminary test was performed. After incubation of the immunosensor in a solution of S-Protein + N-protein, the electrochemical response by DPV showed no variation, with similar values of Ipa for the sample with or without N-protein (Fig. 6B).

Saliva is a type of sample where high concentrations of SARS-CoV-2 virus biomarkers can be detected, and the collection can be performed in a simple and less invasive way for patients, when compared to other samples such as blood or nasopharyngeal samples.^34^ ^,^ ^35^ ^,^ ^36^ Aiming at the immunosensor applicability, real samples were simulated using artificial saliva + S-protein. The antigen quantification using the DPV technique recorded a good correlation, with similar result obtained in standard solution (Table I).

**TABLE I:** Comparison of S-protein concentration in saliva samples by differential pulse voltammetry using the developed immunosensor [average of three determinations + standard deviation (SD) ]

S-protein (ng mL^-1^)	Differential pulse voltammetry
10	10.2 ± 0.05
100	100.8 ± 0.3
250	249.3 ± 0.7
500	499.6 ± 1.4
750	750.9 ± 1.6
1000	1001.7 ± 1.9

Since the outbreak of the disease caused by SARS-CoV-2, several electrochemical biosensors have been reported for application in S-protein detection. These devices have shown excellent performance and low detection limits (Table II). However, the manufacturing methodologies are more complex than those presented in this work, and our results are adequate when compared to bibliography, so it is a good alternative platform that could be considered for Covid-19 diagnosis.

**TABLE II:** Electrochemical biosensors reported for severe acute respiratory syndrome coronavirus 2 (SARS-CoV-2) S-protein determination

Biosensor	Limit of detection	References
S-antibody/1-pyrenebutyric acid N-hydroxysuccinimide ester/Graphene Field-Effect Transistor	1 fg mL^-1^	^12^
S-antibody/1-pyrenebutyric acid N-hydroxysuccinimide ester/graphene electrodes	20 *μg* mL^-1^	^15^
S-antibody/Cysteamine/Interdigitated electrodes	2.5 *μg* mL^- 1^	^13^
S-antibody/Screen-printed carbon electrodes	0.15 ng mL^-1^	^16^
Fc-tag-tagged human ACE2/Cysteamine- Glutaraldehyde/SiO_2_@UiO-66 / Screen-printed carbon electrodes	100 fg mL^-1^	^37^
S-antibody/carboxylated magnetic beads / Screen-printed gold electrodes	22.5 ng mL^-1^	^38^
S-protein+3-aminophenylboronic acid/4-Mercaptophenylboric acid/Cu_7_S_4_-Au/ Screen-printed carbon electrodes	1 copy μL^-1^	^39^
S-protein+3-aminophenylboronic acid/4-Mercaptophenylboric acid/Cu_7_S_4_-Au/ Screen-printed carbon electrodes	1.76 pg mL^-1^	^40^
Methylene blue/DNA aptamer/Gold nanoparticles/Indium Tin Oxide Glasses	91.1 pmo L^-1^	^41^

In conclusion, this experimental study allowed the development of a simple and label-free platform for electrochemical detection of the SARS-CoV-2 S protein. The results demonstrated that Cys SAMs are an easy and stable strategy for the construction of immunosensors. The obtained sensitivity was good, and the method was selective for the target analyte, with better results than the ELISA test. Saliva samples spiked with antigen exhibited similar results to S-protein standards, confirmed no interference that other molecules in the electrochemical response of the immunosensor. This alternative option is promising for the rapid diagnosis of Covid-19.
